# Electricity in Disease

**Published:** 1847-02

**Authors:** 

**Affiliations:** Dublin


*Electricity in Disease.* By Mr. DONAVAN, of Dublin.—The first attempt at rendering electricity available in the alleviation of human suffering was made by Kratzentein, in 1744, who tried it in several diseases. Shortly afterwards, a number of works were published on medical electricity, containing, with some truth, many fictitious and marvellous accounts: all this might be expected.

Signor Pivati, of Venice, published, in Italian, an Essay on Medical Electricity, in which he narrated many wonderful things. He included balsam of Peru in a glass cylinder, so closely that there was not the least smell from it. With this cylinder he electrified a person who had a pain in his side; the man went home, went to bed, slept, sweated, scented the bed, his clothes, and even his hair, to such an extent that even the comb smelt of the balsam. Another person, electrified in a similar manner, became in half an hour more lively and cheerful than usual: the company perceived a smell for which neither he nor they could account, until at length he discovered that it proceeded from himself. By electric exhalations Pivati cured amenorrhœa. He had also the honor and pleasure of curing Donadoni, Bishop of Sebenico, aged seventy-five years, of gout. The Bishop came with his physician and friends; the disease had stiffened his fingers and knees so that he could not bend them; he was obliged to be lifted into bed every night. Pivati filled his cylinder with discutients, and with it electrified his reverend patient. In a few minutes the Bishop felt an unusual commotion in his fingers; he was relieved; he opened and shut both his hands, and gave a hearty squeeze to one of his servants: he rose up, walked, smote his hands together, helped himself to a chair, sat down, walked down stairs with the agility of a young man, and was so amazed that he thought it must be a dream. In the same manner a lady aged 60, with fingers swollen by the gout during six months, always trembling, and with one arm convulsed, was fully relieved by two minutes' application of the medicated electricity.

Pivati proposed to electrify his patients by cylinders filled with medicines of all kinds, as diuretics, antiapoplectics, sudorifics, &c. These medicated cylinders were named *intonacatures.*

The Abbé Nollet was so amazed at these wonderful things that he paid a visit to Pivati in Italy, and questioned him pretty sharply concerning the statements he hed published. Pivati, it appears, could not stand the cross-examination: he confessed to the Abbé that as to the odors he had never transmitted them but twice, although he made many trials. When asked to produce the successful cylinder, he said it was broken, and he could not produce even a bit of it. The Abbé then took him to task about the Bishop of Sebenico, but was informed that the poor bishop was as bad as ever. Several other exposures were also made.

All these wonders, sufficiently startling even to ordinary credulity, were nevertheless, adopted by professor Winkler, of Leipsic, who added a few of his own. He included sulphur, and, in another case, cinnamon, in a glass sphere, electrified it, and filled the room with the vapor of each, which, in the case of the cinnamon, he perceived in his mouth even the next day. Dr. Bianchini's *Recueil d'Experiences faites à Venise* was mainly instrumental in exposing these absurdities.

Dr. Giuseppe Bruni, one of the principal physicians of Turin, about this period reported that at Bologna a man deaf of one ear, having a continued noise in it, and occasionally suffering violent pain, was electrified by Dr. Verati, sparks being taken from the neighborhood of his ear; the parts were reddened as if by a blister; the patient passed the night with less pain and noise, and was perfectly cured of his disorder.

Dr. Bruni then relates some other cases of cure, all of which are quite probable, but he shakes our confidence in his own judgment by stating that a physician at Some lined a cylinder with purgative matter, and electrified a gentleman with it, who immediately found on the spot the same effects as if he had swallowed the cathartic. If this happened, it must have been fright that acted.

The following case is well attested, and deserves attention.

A woman aged 33, whose father had died of paralysis, was attacked with the same disease, which sometimes affected her in the left arm, and sometimes in the leg of the same side; and although these parts lost all power of motion, they retained sensibility; but after a while she lost all sensation of the left side. Becoming worse, her head shook, her tongue faltered, her left eye became so dim that she could not distinguish colors with it; and she was oftened seized with universal coldness and insensibility, so that it was sometimes doubtful whether or not she was dead.

In this state the Rev. Mr. Brydone determined to try electricity. He therefore communicated several very severe shocks, which seemed to raise her spirits; she said she felt a heat and pricking pain in her left thigh and leg, which generally spread over the whole side, and after undergoing the operation a few minutes longer, she "cried out with great joy that she felt her foot on the ground." The electric machine producing such extraordinary effects, the woman submitted to receive above 800 shocks, the consequence of which was, that the shaking of her head gradually lessened, until it entirely ceased; she was able, at last, to stand without support, and on leaving the room forgot one of her crutches. Next day, being electrified as before, her strength sensibly increased; she walked easily without a stick, and could lift several pounds weight with the hand that had been paralytic. The experiment was repeated on the third day, by which time she received, in all, upwards of 600 severe shocks, when, declaring herself to have as much power on the left side as the right, she was pronounced cured. This account was attested by herself, by the minister of Coldingham, in Berwickshire, and by a letter from Dr. Whytt to Dr. Pringle.

Professor Imhoff, of Munich, thus generalises the effects of electricity: It promotes the free circulation of the fluids, and especially of the blood; it accelerates perspiration, increases animal heat, and promotes all the secretions and excretions of the body. The essential condition of the useful application of frictional electricity is that the machine employed shall be one of considerable power, although its utmost intensity is by no means to be always brought to bear on the patient. When the rubefacient effects, or the aura, or the stream, or the local stimulus of electricity are required, they can only be obtained from the largest and most powerful machine.

The latest testimony on record of the medical efficacy of frictional electricity is to be found in a report of Dr. Golding Bird, who declares that scarcely any cases have been submitted to electrical treatment in which its sanatory influence has been so strongly marked as those in which the menstrual function was deficient. While chlorosis is present, scarcely any benefit is to be expected. The rule for insuring success, in the great mass of cases of amenorrhœa, is to improve the general health by exercise and tonics, and to remove accumulations in the bowels; a few electric shocks, often a single one, will then be sufficient to produce menstruation. The shock of a quart Leyden jar is to be passed from the pubes to the sacrum, as often as necessary, beginning about a week before the expected return of the menstrual discharge.

